# Character Decomposition and Transposition Processes in Chinese Compound Words Modulates Attentional Blink

**DOI:** 10.3389/fpsyg.2016.00923

**Published:** 2016-06-20

**Authors:** Hongwen Cao, Min Gao, Hongmei Yan

**Affiliations:** ^1^Key Laboratory for NeuroInformation of Ministry of Education, University of Electronic Science and Technology of ChinaChengdu, China; ^2^Center for Information in BioMedicine, University of Electronic Science and Technology of ChinaChengdu, China

**Keywords:** Chinese compound word, character decomposition, character transposition, attentional blink, rapid serial visual presentation

## Abstract

The attentional blink (AB) is the phenomenon in which the identification of the second of two targets (T2) is attenuated if it is presented less than 500 ms after the first target (T1). Although the AB is eliminated in canonical word conditions, it remains unclear whether the character order in compound words affects the magnitude of the AB. Morpheme decomposition and transposition of Chinese two-character compound words can provide an effective means to examine AB priming and to assess combinations of the component representations inherent to visual word identification. In the present study, we examined the processing of consecutive targets in a rapid serial visual presentation (RSVP) paradigm using Chinese two-character compound words in which the two characters were transposed to form meaningful words or meaningless combinations (reversible, transposed, or canonical words). We found that when two Chinese characters that form a compound word, regardless of their order, are presented in an RSVP sequence, the likelihood of an AB for the second character is greatly reduced or eliminated compared to when the two characters constitute separate words rather than a compound word. Moreover, the order of the report for the two characters is more likely to be reversed when the normal order of the two characters in a compound word is reversed, especially when the interval between the presentation of the two characters is extremely short. These findings are more consistent with the cognitive strategy hypothesis than the resource-limited hypothesis during character decomposition and transposition of Chinese two-character compound words. These results suggest that compound characters are perceived as a unit, rather than two separate words. The data further suggest that readers could easily understand the text with character transpositions in compound words during Chinese reading.

## Introduction

The human visual system has developed a remarkable capacity to process sequential perceptual information. However, there are clear limitations to human conscious perception, as evidenced by the attentional blink (AB). The AB refers to an observer’s attenuated ability to report the second of two targets (T2) in a rapid serial visual presentation (RSVP) stream if it appears within 500 ms after the first target (T1). However, there is little or no performance deficit if T2 is presented immediately after T1, a phenomenon known as “Lag 1 Sparing” (Potter et al., 1998). AB is a well-established technique for investigating the temporal properties of visual perception (Potter, 1976; Raymond et al., 1992; Chun and Potter, 1995; Visser et al., 1999).

For two decades, there has been a substantial research effort dedicated to the underlying cause of this robust attentional phenomenon (Dux and Marois, 2009; Martens and Wyble, 2010). The associative relationship between T1 and T2 is one of the major contributors to the magnitude of AB. Priming is determined by the degree of decreased impairment in the magnitude of the AB when T1–T2 are related (Maki et al., 1997; Potter et al., 2005), and the priming effect occurs regardless of the stimulus onset asynchrony (SOA) between T1 and T2 (Juola et al., 2000) or whether the target shown during the AB task is identified (Martens et al., 2002).

Potter et al. (2005) claimed that T1 is primed only with short SOAs, whereas T2 is primed with longer SOAs or any SOA. They also proposed that priming is functionally unidirectional at very short SOAs (<100 ms), from the first identified word to the second word not yet identified (Potter et al., 2005). Therefore, if T2 is identified first, it will prime the identification of T1 when T1 and T2 are related. However, if the two words are unrelated, neither target benefits. At longer SOAs, T1 is almost always identified and consolidated first, and T1 only acts as a prime for T2 in a related context.

When T2 is identified first, errors in reporting order may occur. Specifically, observers may reverse the temporal order of the two targets—T1 is reported as T2, and T2 is reported as T1 (Lawrence, 1971; Chun and Potter, 1995; Spalek et al., 2006, 2012; Akyürek et al., 2007). The proportion of order reversals shows a substantial decrement from Lag 1 to Lag 3 because the loss of episodic distinctiveness leads to order confusion at Lag 1, and the two events are integrated within one attentional episode. As the SOA between T1 and T2 is increased, the temporal discriminability of the two targets increases correspondingly. Therefore, the prevalence of order errors can be used as a measure of joint integration (Akyürek et al., 2007).

It must be noted that the perception of the temporal order in AB has been explored using English letters, symbols, and numbers (Chun and Potter, 1995; Olivers et al., 2011; Akyürek et al., 2012; Hilkenmeier et al., 2012; Spalek et al., 2012), but such studies have rarely used Chinese characters and words as stimuli. Logographic Chinese differs markedly from alphabetic English. English words are composed of letters, whereas Chinese words are composed of characters. Strings of these characters form Chinese words and text. In our previous study, we varied the SOAs and the orthographic, phonological, semantic, and lexical connections between two Chinese characters. We found that AB is hierarchically attenuated in T1–T2 pairs that were related phonologically, morphologically, or semantically, but the effect of AB was absent when T1 and T2 were a two-character compound word (Cao et al., 2014). Thus, priming plays an important role in the hierarchical modulation of AB.

Moreover, the well-known “Cambridge University effect” demonstrated that jumbled letters (letter randomization in the middle of the word) had little effect on the ability of skilled readers to understand the printed text (Davis, 2003). Some masked priming and transposition priming studies of this effect have suggested the reading processing system in humans is a fast, automatic, and comparably robust system, but it is also affected by factors that include the letter space, relative position, and lexical organization (Grainger and Whitney, 2004; Rayner et al., 2006). An investigation of Hebrew–English bilinguals even showed that the effects of letter transposition on reading are language specific (Velan and Frost, 2007). Chinese consists of a large number of two-character compound words (approximately 72%; Language and Teaching Institute of Beijing Linguistic College, 1986). As special linguistic characteristics in Chinese, these constituent components in compound words are separable, and their positions are also flexible. The combination of two-character compound canonical words can generate two types of corresponding words by varying the order of the components. These words include the reversible word and the transposed word, depending on whether they are lexically meaningful. For example, the word “
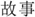
” (‘story’), which is made up of two components (“
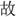
” ‘old’ and “
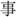
” ‘matter’), can be reversed and still constitutes a meaningful word “
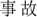
” (‘accident’). This unique compound is referred to as a ‘reversible word.’ The transposed word “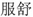,” which is obtained by reversing the constituent morphemes of the canonical two-character word “
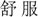
” (‘comfortable’), shares visual similarity with its corresponding canonical characters, but the character position is violated and the transposed word is meaningless. Several studies using a lexical decision paradigm (Taft et al., 1999; Liu et al., 2010; Bai et al., 2011) or masked priming paradigm (Gu et al., 2015) have demonstrated that Chinese words show insensitivity to the positional information of the constituent morphemes, which is similar to the hypothesis that the letter order is not strictly encoded in alphabetic scripts (Rayner et al., 2006; Johnson and Eisler, 2012). However, previous experiments have not investigated the temporal dynamics of Chinese character order encoding or the effects of word integration during AB, especially for two-character Chinese compound words. In fact, the flexible positions between Chinese two-character compound words (I. canonical words, II. transposed words, and III. reversible words) provide an effective method for examining the temporal course for the priming of AB, as well as the effects of character transposition and lexical organization on reading.

In the present study, we focus on answering the following two questions: (1) whether the decomposition and transposition of constituent characters in compound words attenuates or eliminates the magnitude of AB. (2) How the compound words and their position information are processed in the AB paradigm. We investigated the above questions using a classical AB experimental procedure in which pairs of Chinese characters in four different categories were presented as stimuli.

## Materials and Methods

### Participants

Twenty native Chinese speakers (10 females and 10 males, aged 22–30, Mean = 25.8, *SD* = 2.61) with normal or corrected-to-normal vision participated in the study. All subjects provided written informed consent prior to participation. The experimental paradigms were approved by the Ethics and Human Participants in Research Committee at the University of Electronic Sciences and Technology of China in Chengdu, China. All subjects were blinded to the purpose of the experiment and were familiarized with the task by performing an initial training of 40 trials before the experimental phase began.

### Apparatus

The tasks were performed in a sound-attenuated room that was specially designed for psychophysics experiments, and the room illumination was maintained at the same level for all participants. The stimuli were presented on a high-resolution (1024 × 1280 pixels) computer monitor with a refresh rate of 100 Hz. The Chinese characters used in the experiments appeared on the center of a gray background that was adjusted to a mean luminance of 9.1 cd/m^2^. The stimulus presentation program was compiled in MATLAB (MathWorks, Natick, MA, USA) using Psychtoolbox (Brainard, 1997; Pelli, 1997).

### Stimuli

**FIGURE 1:**
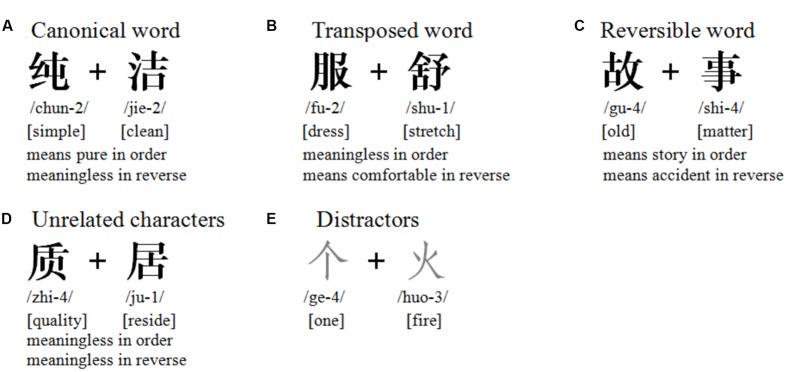
**Examples of the four categories of experimental stimuli pairs and distractors.** **(A)** Canonical word, **(B)** transposed word, **(C)** reversible word, **(D)** unrelated characters, **(E)** distractors. The pronunciation and word meanings of each constituent morpheme of the compound words are listed below the characters; the number following pinyin denotes the tone.

The target stimuli T1 and T2 consisted of paired two-character Chinese compound words in four categories. (1) For canonical words, e.g., “
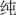
” (simple, T1) and “
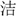
” (clean, T2), the two targets (T1 + T2) form a unidirectional two-character Chinese compound word. In the above example, the characters mean “pure” when they are written together in order, but they are meaningless when they are written in the reverse order. (2) Transposed words are meaningless when they are written together in order and are obtained by reversing the constituent morphemes of a unidirectional canonical two-character word, e.g., “
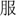
” (dress, T1) and “
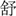
” (stretch, T2). In this example, the pseudo-phrase (T1 + T2) is obtained by transposing the order of the constituent morphemes of the canonical word “
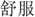
” (T2 + T1). (3) Reversible words, e.g., “
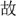
” (old, T1) and “
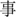
” (matter, T2), are bidirectional-words (T1 + T2, means story) that are still meaningful when the character order is reversed (T2 + T1, means accident). (4) For unrelated characters, e.g., “
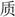
” (quality, T1) and “
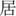
” (reside, T2), the two characters form a meaningless pseudoword regardless of their order. The four categories of character combinations are shown in **Figure 1.**

The selected two-character compound words were commonly used and had frequencies of occurrence of no fewer than 40 per million. According to the Language and Teaching Institute of Beijing Linguistic College (1986), the mean frequency was 76.21 (*SD* = 14.48) for canonical words, 71.83 (*SD* = 17.47) for their corresponding transposed words, and 79.32 (*SD* = 12.77) for reversible words. The mean single-word frequency of the first and second characters were 82.73 (*SD* = 10.88) and 82.11 (*SD* = 11.40) for canonical words, 80.14 (*SD* = 13.28) and 79.42 (*SD* = 12.95) for transposed words, 75.37 (*SD* = 13.46) and 72.92 (*SD* = 15.65) for reversible words, and 75.97 (*SD* = 16.06) and 74.43 (*SD* = 17.49) for unrelated characters, respectively. The visual complexity of the compound characters was matched across each stimulus type. The average number of strokes in the T1 and T2 characters was 9.71 (*SD* = 2.58) and 9.51 (*SD* = 2.48) for canonical words, 9.32 (*SD* = 2.21) and 9.48 (*SD* = 2.69) for transposed words, 8.41 (*SD* = 2.8) and 8.55 (*SD* = 3.01) for reversible words, and 8.01 (*SD* = 2.37) and 8.03 (*SD* = 2.28) for unrelated characters, respectively. There were no significant differences in strokes and word frequencies between T1 and T2 among the four conditions (*p* > 0.05 in all cases).

Each category included 128 stimulus pairs, resulting in a total of 512 pairs of Chinese words. The distractors consisted of 100 of the most frequently used Chinese characters (2–9 strokes). These distractors were totally irrelevant to the targets in terms of their semantic or lexical information. **Figure 1** contains an example from each of the four categories of experimental character pairs, as well as the distractors. The English translations are listed underneath the Chinese characters.

Each character was displayed on the screen with the same size (0.86° × 0.95°). The stimulus pairs and distractors were randomly chosen for each trial. The characters chosen as T1 and T2 for the discrimination task were displayed in bold, whereas the distractors were presented in a normal font.

### Procedure

**FIGURE 2:**
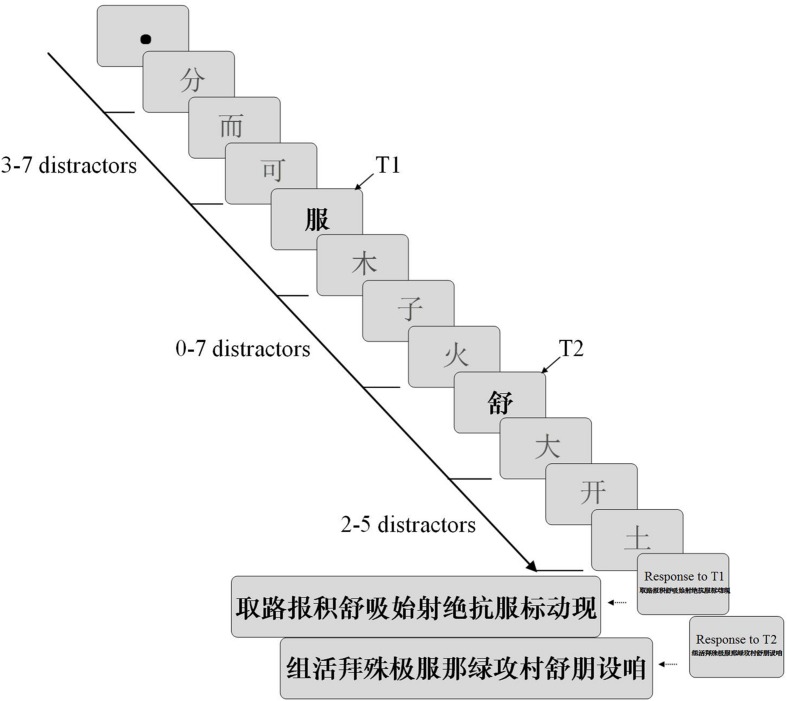
**Schematic illustration of the sequence of events within a trial.** The target characters were presented in bold black font, and the distractors were displayed in normal font. The serial position of targets between T1 and T2 were systematically varied in the sequence. After the stream was presented, participants were instructed to identify the two targets. The time course of interference was measured as a function of the temporal separation between the targets (SOA).

Participants were tested with a viewing distance of 60 cm, and head movements were prevented during the experiment by immobilizing the head in a fixed position using forehead and chin rests. In each trial, a fixation dot (0.3° in diameter) was presented for 800 ms. Then, an RSVP stream of Chinese characters were presented sequentially at a rate of 60 ms/item in the center of the screen. Participants were instructed to identify the two bold black Chinese characters (referred to as targets marked T1 and T2) embedded in the normal font Chinese character stream. The number of distractors appearing prior to T1 were chosen at random and varied from 3 to 7. The position of T1 was randomly permuted in serial positions 4–8. There were eight SOAs between T1 and T2 from Lag 1 (no intervening items, SOA = 60 ms) to Lag 8 (SOA = 480 ms) and these SOAs were systematically varied. Finally, at least 2–5 items followed T2, signaling the end of the stream. After the rapid succession, the first panel containing 14 bold black Chinese characters was displayed on the screen. The subjects were asked to identify the T1 embedded in the stimuli by clicking on it with a computer mouse. The subjects’ response was not timed. Once T1 was selected, a second panel with another 14 characters was presented to identify T2. **Figure 2** shows the sequence of events in a typical trial.

Participants initiated the next trial by pressing the space bar on the keyboard. The items presented in a given trial were randomly generated with the condition that no character was presented twice within a trial. All the target items were presented only once in the experiment. Subjects were asked to not wildly guess when attempting to select the correct characters. Each subject performed two sessions, and each session included four blocks of 256 trials, resulting in a total of 512 individual trials. The types of stimuli pairs and the lags (SOAs) were presented randomly in each block.

### Statistical Analysis

Analyses of variance (ANOVA) were performed by subject (*F*_1_) and by item (*F*_2_) to test differences among the four stimulus categories. Mean accuracies and proportions of order errors were analyzed by means of two-way repeated-measures ANOVAs using Word category (unrelated, transposed, canonical, and reversible word conditions) and the SOA (60, 120, 180, 240, 300, 360, 420, and 480 ms) as between- and within-subjects factor. A significance level of *p* < 0.05 was adopted for all tests.

## Results

**FIGURE 3:**
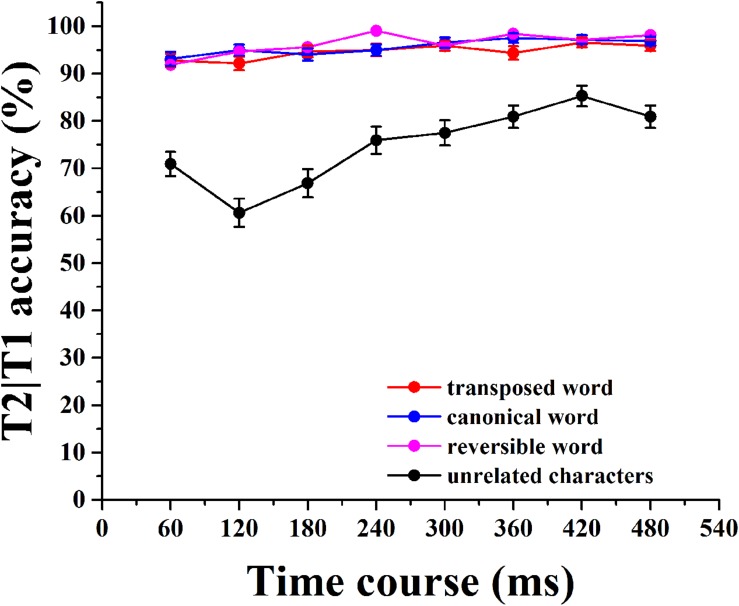
**Mean accuracy of T2 given accurate identification of T1 (T2|T1) across all SOAs in each of the four conditions.** Error bars represent the standard error.

We analyzed the percentage of correct T2s identified from trials in which T1 was accurately identified (T2|T1) as a function of the SOA of T1–T2. Targets were scored as correct regardless of the order of identification (Chun and Potter, 1995). The mean percentages of T2|T1 were computed for each subject at each SOA, and they were calculated for each stimulus category. **Table 1** contains the average percentage of trials for only T1; only T2 and T2|T1 were accurately identified in the four conditions. The average accuracies for T1 and T2 for the transposed, canonical, and reversible conditions were all good (above 97%). The mean performances for identifying T2|T1 in unrelated, transposed, canonical, and reversible word conditions were 74.84, 94.69, 95.66, and 96.37%, respectively. A standard AB deficit was obtained in the unrelated condition [*F*_1(7,133)_ = 9.76, *p* < 0.001, $\eta_{p}^{2}$ = 0.05, *F*_2(7,3577)_ = 11.83, *p* < 0.001, $\eta_{p}^{2}$ = 0.03], and the performance for T2|T1 was impaired when T2 was presented shortly after T1. However, the accuracy in identifying T2|T1 was almost identical across all SOAs under the transposed, canonical, and reversible word conditions (all *p* > 0.05), indicating the AB was absent (**Figure 3**). These results revealed that the reversibility and transposition of the characters within a compound word contributed to the elimination of AB.

**Table 1:** Mean accuracy in reporting T1, T2, and T2|T1 in the four stimulus categories during an attentional blink paradigm.

Category type				
	
	Unrelated	Transposed	Canonical	Reversible
T1	83.09	97.19	98.01	97.49
T2	88.94	97.34	97.77	98.87
T2|T1	74.84	94.69	95.66	96.37


The conditional T2 accuracy was also analyzed using a 4 (category) × 8 (SOA) repeated-measures ANOVA. A main effect of category was obtained [*F*_1(3,57)_ = 113.73, *p* < 0.001, $\eta_{p}^{2}$ = 0.35, *F*_2(3,1533)_ = 163.35, *p* < 0.001, $\eta_{p}^{2}$ = 0.28], and a main effect of SOA was also found [*F*_1(7,133)_ = 18.82, *p* < 0.001, $\eta_{p}^{2}$ = 0.03, *F*_2(7,3577)_ = 19.23, *p* < 0.001, $\eta_{p}^{2}$ = 0.02]. The interaction between these two factors was significant [*F*_1(21,441)_ = 6.42, *p* < 0.001, $\eta_{p}^{2}$ = 0.03, *F*_2(21,10731)_ = 6.56, *p* < 0.001, $\eta_{p}^{2}$ = 0.02]. The results of the *post hoc* multiple comparisons revealed significant differences for the transposed, canonical, and reversible word conditions compared with the unrelated characters condition (*p* < 0.001 in all cases), whereas there were no significant differences among the three word categories (all *p* > 0.05). The three similar patterns of results demonstrated an overall associative boost across SOAs under the transposed, canonical, and reversible word conditions. For the transposed condition, the transposition of two characters within the compound word provided a reverse priming to the word identification, reflecting the encoding of character order. However, the transposition of the characters in compound words did not affect the visual word processing in Chinese reading. These results revealed that the three categories of compound words were processed as a whole, even when presented in transposed order. The characters in compound words were read simultaneously, and the readers could correct minor errors in character order according to the representation of the original word in long-term memory.

**FIGURE 4:**
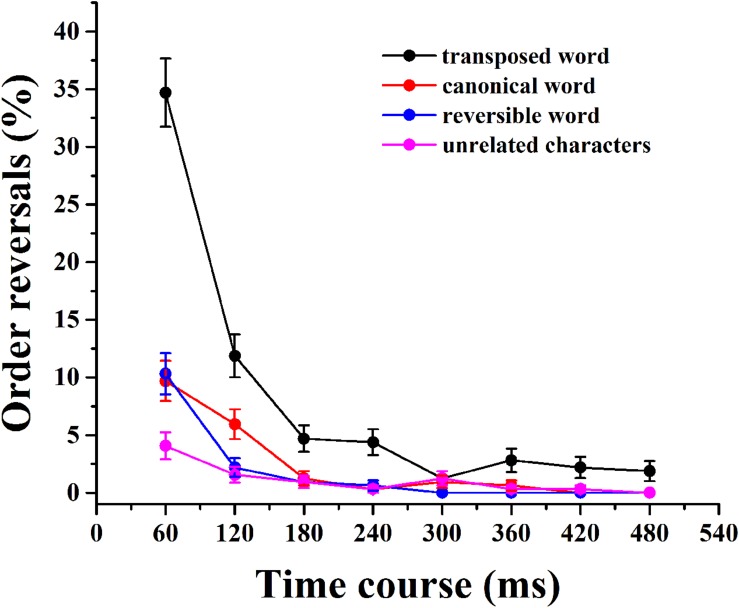
**The proportion of order reversals for the four categories.** Error bars indicate the standard error.

As described in the introduction, the competition between the two targets may lead to errors in the reporting order for the AB at short SOAs. **Figure 4** shows the percentages of the transposition probability of the character order for the four categories of stimuli pairs. The subjects had a higher chance of reporting T2 as T1 and T1 as T2 when the SOAs were extremely short, the same order of its corresponding original words in canonical, transposed, and reversible word conditions. Although similar tendencies toward order reversal were observed for unrelated word conditions, the amplitudes were much weaker. A 4 (category) × 8 (SOA) repeated-measures ANOVA was carried out to reveal the transposition significance of the character order across the four categories. There were significant main effects of category [*F*_1(3,57)_ = 61.81, *p* < 0.001, $\eta_{p}^{2}$ = 0.23, *F*_2(3,1533)_ = 76.54, *p* < 0.001, $\eta_{p}^{2}$ = 0.15] and SOA [*F*_1(7,133)_ = 104.22, *p* < 0.001, $\eta_{p}^{2}$ = 0.14, *F*_2(7,3577)_ = 112.62, *p* < 0.001, $\eta_{p}^{2}$ = 0.08]. More importantly, the interaction between the two factors was also significant [*F*_1(21,441)_ = 21.87, *p* < 0.001, $\eta_{p}^{2}$ = 0.09, *F*_2(21,10731)_ = 23.63, *p* < 0.001, $\eta_{p}^{2}$ = 0.05]. These results showed that the order reversal patterns varied across SOAs and categories. The fact that the perceived order of the two characters was often reversed at very short SOAs suggested some competition and loss of episodic distinctiveness between the targets. It should be noted that this order reversal was only very obvious and longer-lasting in transposed words, while this effect was very weak and limited mostly to Lag 1 (only canonical word might extend to Lag 2) in all the other three types. The highest proportion of order reversals at each Lag 1 revealed that the two targets might be integrated within a single representation episode. This result indicated that the two targets had a comparably high percentage chance to be identified, and the target identified first primed the identification of the second target in the early SOAs. The earlier the SOA, the more obvious the order reversal, and the more obvious the mutual priming between T1 and T2. The results of the *post hoc* multiple comparisons revealed significant differences for the unrelated characters, as well as the canonical and reversible word conditions compared with the transposed word condition (*p* < 0.001 in all cases). The greatest surplus of reversals in the transposed condition occurred at an SOA of 60 ms, which then dropped precipitously, suggesting the strongest order competition between the two targets that leaded to the loss of episodic distinctiveness.

## Discussion

The present results indicate that the relation priming of Chinese two-character compound words contributes to the elimination of the AB, regardless of the character order within the compound word. These character decompositions and transpositions in Chinese compound words had priming effects on word processing across all SOAs. The data further demonstrates that readers have the inclination to correct some minor errors in the character order coding of compound words and integrate them as a unit during reading.

The question addressed here is whether the AB is a resource limitation or a cognitive strategy during character decomposition and transposition of Chinese compound words. The former claims that the occurrence of the AB is due to the allocation of limited attentional resources during two-target processing (Chun and Potter, 1995; Isaak et al., 1999; Jolicœur and Dell’Acqua, 1999). The latter argues that working memory encoding of T1 suppressed the deployment of attention to T2 in order to prevent the perceptual inputs from being integrated with the episodic memory of T1 (Wyble et al., 2009). This account suggests a cognitive strategy of enforcing the episodic distinction between the two targets, and when the memory representations of separately presented targets lack episodic distinctiveness, the target items are identified in an incorrect order. The results obtained in the present study provide support for the latter account. Although, observers can identify the successive target characters under all three word conditions, they have problems accurately differentiating the order between the characters presented first and second. The two targets are jointly encoded into working memory when they are presented in immediate succession regardless of their order. This loss of episodic distinctiveness causes the temporal order of the two targets to often be reversed at very short SOAs. Note that this order reversal was only very obvious and longer-lasting in transposed words. In all the other three types, this effect was very weak and limited mostly to Lag 1 (only canonical word might extend to Lag 2; **Figure 4**). The present results are therefore more consistent with the cognitive strategy hypothesis (Wyble et al., 2009) than the resource-limited hypothesis (Chun and Potter, 1995) during Chinese two-character word identification.

A general question regarding relation priming for the two targets is whether it is functionally unidirectional or bidirectional in AB. Chun and Potter (1995) found that the perceived order is usually reversed when the two targets are presented in close temporal succession. The two-stage competition model claims that a strong competition exists between two target characters in the early stages of processing, with T2 being identified prior to T1 at short SOAs, typically within 200 ms, thus priming T1. Moreover, this competition is mutual but also an effect of T1 on T2 as the SOA increases to 213 ms (Potter et al., 2002, 2005). Our results revealed that the first identified character (T1 or T2) increased the probability of identifying the second target (T2 or T1), and such relation priming was mutual for the two targets at very short SOAs in transposed, canonical, and reversible conditions (**Figure 4**). The performance for canonical words was almost identical across all SOAs, which was consistent with our previous study that involved lexical-semantic priming with two-character compound words (Cao et al., 2014). The similar patterns of results across three word categories indicate that the bidirectional relation priming between T1 and T2 boosts compound word processing across all SOAs and contributes to the elimination of AB.

There may also be other interpretations of the same results. The almost identical patterns of results across the three word conditions suggest that two-character compound words are processed as a whole, regardless of the order of the characters in the compound word. Moreover, this whole-word level of representation occurs regardless of the SOA between the two target characters. The highest overall transposition probability of the character order in transposed words indicates the holistic representation of the original word is activated. Additionally, the components of compound words contribute to whole-word processing. Zhou et al. (1999) proposed that the processing of the initial morphemes activated the semantic representations of whole words, and this semantic activation served as a contextual constraint in interpreting the ambiguous second character. Our results demonstrated that the semantic context set up by the initial constituent morpheme, transposed or not, consistently assisted identification of the other character across all SOAs.

Prior studies have demonstrated that some letter transpositions in words have little or no effect on the ability of skilled readers to understand the text in the alphabetic writing system (Davis, 2003; Grainger and Whitney, 2004; Rayner et al., 2006). Our results revealed that we could easily identify the transposed words across all the SOAs. Specifically, the character order information was not strictly processed during two-character compound word processing. The highest proportion of order reversals in the transposed word condition revealed the modulation of top-down information in the mental lexicon when the bottom-up input showed an inconsistency between the transposed and original words. Characters in compound words are read simultaneously, and the human brain has the inclination to automatically correct minor errors according to the holistic representation of the canonical words in long-term memory.

## Conclusion

The AB occurred when two characters could not be integrated into a single compound word (pseudoword condition); however, such an effect was modulated by the character decomposition and transposition processes in Chinese two-character compound words. Specifically, the ABs were eliminated when two characters could be integrated into a single compound word regardless of their orders. The present results are therefore more consistent with the cognitive strategy hypothesis (Wyble et al., 2009) than the resource-limited hypothesis of Chun and Potter (1995). Additionally, the two-character compound word could be recognized as a unit in the dual-target RSVP tasks, regardless of the order and SOA between the constituent characters within a compound word.

## Author Contributions

Conceived and designed the experiments: HY and HC. Performed the experiments: HC and MG. Analyzed the data: HC and MG. Contributed reagents/materials/analysis tools: HC. Wrote the paper: HC and HY.

## Conflict of Interest Statement

The authors declare that the research was conducted in the absence of any commercial or financial relationships that could be construed as a potential conflict of interest.
